# Recombinant human bone morphogenetic protein-2 inhibits gastric cancer cell proliferation by inactivating Wnt signaling pathway via c-Myc with aurora kinases

**DOI:** 10.18632/oncotarget.11969

**Published:** 2016-09-12

**Authors:** Kwang Bok Lee, Hua Jin, Shuai Ye, Byung Hyun Park, Soo Mi Kim

**Affiliations:** ^1^ Department of Orthopaedic Surgery, Chonbuk National University Medical School, Research Institute of Clinical Medicine of Chonbuk National University-Biomedical Research Institute of Chonbuk National University Hospital, Jeonju, 561-180, Republic of Korea; ^2^ Department of Physiology, Chonbuk National University Medical School, Jeonju, 561-180, Republic of Korea; ^3^ Department of Biochemistry, Chonbuk National University Medical School, Jeonju, 561-180, Republic of Korea

**Keywords:** gastric cancer cell, rhBMP-2, Myc, AURKA, AURKB

## Abstract

The detailed molecular mechanisms and safety issues of recombinant human bone morphogenetic protein-2 (rhBMP-2) usage in bone graft substitution remain poorly understood. To investigate the molecular mechanisms underlying the function of rhBMP-2 in gastric cancer cells, we used microarrays to determine the gene expression patterns related to the effects of rhBMP-2. Based on a gene ontology analysis, several genes were upregulated during the regulation of the cell cycle and BMP signaling pathway. *MYC* was found to be significantly decreased along with its downstream target genes, the aurora kinases (*AURKs*), by rhBMP-2 in the network analysis. We further confirmed this finding with western blot data that rhBMP-2 inhibited c-Myc, AURKs, and β-catenin in SNU484 and SNU638 cells. An AURK inhibitor significantly decreased c-Myc expression in gastric cancer cells. Combination treatment with rhBMP-2 and AURK inhibitor resulted in significantly decreased c-Myc expression compared with gastric cancer cells treated with an rhBMP-2 or AURK inhibitor, respectively. Similar effects for decreased c-Myc expression were observed when we silenced β-catenin in gastric cancer cells. These results indicate that rhBMP-2 attenuated the growth of gastric cancer cells via the inactivation of β-catenin via c-Myc and AURKs. Therefore, our findings suggest that rhBMP-2 could be safely used with patients who undergo gastric or gastroesophageal cancer surgery.

## INTRODUCTION

Gastric cancer is the fourth most common type of cancer worldwide and ranks second in cancer mortality [1]. Growing evidence shows that the incidence of gastric cancer has decreased, but the mortality rate has steadily increased over the past decades because it often presents at an advanced stage with an overall survival of about 10 months [2–5]. The most effective curative treatment for gastric cancer is surgery, with chemotherapy and radiation therapy most commonly recommended after surgery [5–7]. However, long-term survival after current chemotherapy-based treatments for advanced gastric cancer is disappointing [7–9]. Therefore, innovative therapeutic approaches must be explored to improve clinical outcomes for gastric cancer patients.

Current approaches for reconstructive techniques include tissue engineering [10]. Recombinant human bone morphogenetic protein (rhBMP)-2 is commonly used in bone graft substitutions, including for bone fracture and spinal surgery [11]. Moreover, rhBMP-2 enhances osteoinductive factors and improves bone therapy [11–13]. However, the use of rhBMP-2 in the area of a resected tumor or in patients undergoing treatment for malignancy is limited due to the unknown effects of BMP-2 on malignancies and other contraindications [14–16]. Moreover, the function of BMP-2 raises the possibility of cancer promotion since BMP-2 and its receptors are highly expressed in many human cancers [17]. For instance, Kokorina et al. reported that rhBMP-2 treatment induces cancer cell growth and tumor cell invasion [10, 14, 18]. In addition, a study that evaluated the association between rhBMP-2 and malignancy by reviewing 515 articles [19] found that 43 studies indicated that BMP-2 enhances cancer growth. However, 18 studies found that BMP-2 suppresses cancer proliferation [19]. Our recent publications have found evidence of an antitumor effect of BMP-2 in esophageal cancer cells through the activation of the hippo signaling pathway [20] and that rhBMP-2 has an anticancer effect *in vitro* and *in vivo* in breast cancer cell lines [21]. Thus, the use of rhBMP-2 raises safety issues regarding cancer risk, but data on the effect of exogenous BMP-2 on cancer are conflicting. Further studies on BMP-2 and cancer are required.

There have been no reports of a correlation between rhBMP-2 effects and human gastric cancer cells or the use of rhBMP-2 for reconstructive surgery on bone defects by cancer of the gastrointestinal tract. Therefore, the objective of this study was to investigate gene expression related to the mechanisms of rhBMP-2 in human gastric cancer cells. We demonstrated that rhBMP-2 significantly inhibited gastric cell viability, and the effects were mediated by suppressing the expression of β-catenin, c-Myc, and AURKs. These results indicated that rhBMP-2 suppresses activation of the Wnt signaling pathway via c-Myc and AURKs, which may, in part, induce cell death of the gastric cancer cells.

## RESULTS

### Effects of rhBMP-2 on the proliferation of gastric cancer cells

**Figure 1:**
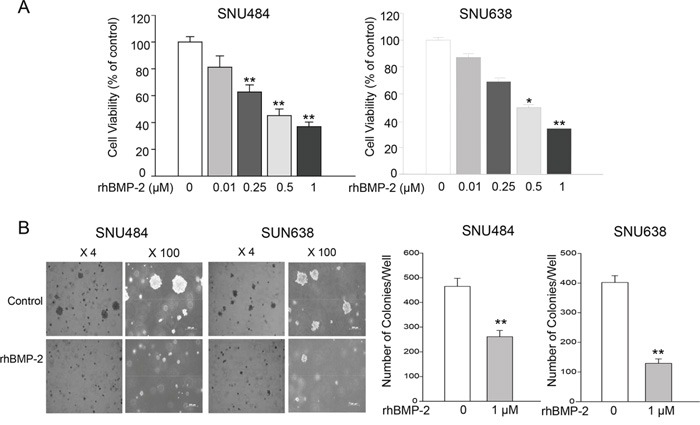
Effects of rhBMP-2 on SNU484 and SNU638 cell proliferation and colony formation **A.** RhBMP-2 inhibited cell proliferation in a dose-dependent manner. **B.** Consistent with MTT assays, significantly fewer colonies were formed compared with the control cancer cells in the presence of 1 μM rhBMP-2 after 30 days. Values represent the mean ± SEM of at least three independent experiments with triplicate plates. ^*^P < 0.05, ^**^P < 0.01 vs. untreated cells.

To investigate the effects of rhBMP-2 on gastric cancer cell proliferation, MTT assays were performed on SNU484 and SNU638 cells. The viability of the SNU484 and SNU638 cells was significantly inhibited following rhBMP-2 treatment in a dose-dependent manner compared with the non-treatment group (Figure 1A). In the SNU484 cell line, inhibition of cell viability with treatment compared to the control group was 81.21% ± 8.50% (P = 0.058) with 10 nM, 62.72% ± 5.31% (P = 0.002) with 250 nM, 45.15% ± 4.91% (P = 0.000) with 500 nM, and 36.95% ± 0.24% (P = 0.000) with 1000 nM BMP-2. In the SNU638 cell line, treatment with same doses of rhBMP-2 resulted in 87.13% ± 4.36% (P = 0.100), 69.01% ± 5.86% (P = 0.029), 49.71% ± 4.15% (P = 0.009), and 34.00 ± 2.97% (P = 0.004), respectively for the inhibition of cell viability compared to the controls. These results demonstrated that rhBMP-2 exhibited significant cytotoxicity on the gastric cancer cells.

### Effects of rhBMP-2 on SNU484 and SNU638 colony formation

Colony formation assays analyzed with the anchorage-independent growth of SNU484 and SNU638 cells in semisolid medium. A significant decrease was observed for the number of colonies of SNU484 and SNU638 cells compared with the control cancer cells after four weeks in the presence of 1 μM rhBMP-2 (Figure 1B). Therefore, RhBMP-2 effectively inhibited the colony formation of gastric cancer cells. These findings confirmed that BMP-2 significantly inhibited gastric cancer cell proliferation by soft agar colony formation assays.

### Effects of BMP-2 on p-Smad1/5/8 expression

**Figure 2:**
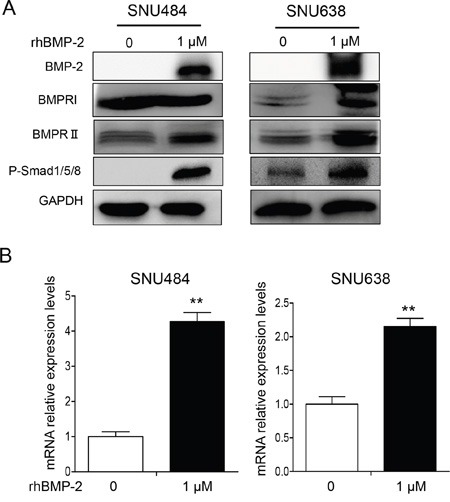
Western blots showing the effect of BMP-2 on Smad signaling pathway-related proteins in SNU484 and SNU638 cells **A.** BMPRI, BMPRII, and p-Smad1/5/8 were measured by western blots of SNU484 and SNU638 cells after treatment with rhBMP-2 for 72 h and immunoblotting with the indicated antibodies. GAPDH was used as an internal control. **B.** Endogenous BMP-2 mRNA determined by qRT-PCR after treatment of SNU484 and SNU638 cell lines with rhBMP-2 for 72 h. The ratios of the normalized expression of the target genes relative to the control (untreated) cells are shown. Results are the means ± SEM. β-actin was used as an internal control. ^**^P < 0.01 vs. untreated cells.

We next investigated whether rhBMP-2 increased bone morphogenetic protein receptor (BMPR) I, BMPRII, and p-Smad1/5/8 proteins. Expression of rhBMP-2 protein significantly increased following treatment with rhBMP-2 (Figure 2A). To address whether the treatment with rhBMP-2 in gastric cancer cells could increase the level of endogenous BMP-2 expression, we performed real-time RT-PCR to measure the endogenous *BMP-2* expression following treatment with rhBMP-2 in SNU484 and SNU638 cells. We found that rhBMP-2 significantly increased *BMP-2* mRNA levels in SNU484 and SNU638 cells (Figure 2B). RhBMP-2 increased both the mRNA and protein levels of BMP-2 in gastric cancer cells, which is in line with the microarray analysis of the activation of the BMP-2 signaling pathway. We measured ERK1/2 and p-ERK1/2 expression following treatment of rhBMP-2 in gastric cancer cells. RhBMP-2 suppressed p-ERK1/2 expression in SNU484 and SNU638 cells at 48 h following treatment of rhBMP-2, while ERK1/2 expression remained unchanged (Supplementary Figure S1). The BMPRII protein levels increased and p-Smad1/5/8 protein expression was significantly increased following rhBMP-2 treatment of SNU484 and SNU638 cells. Therefore, rhBMP-2 appeared to stimulate the activation of BMPRII, which stimulated the expression of p-Smad1/5/8 proteins in gastric cancer cells. Furthermore, Smad signaling was induced by the rhBMP-2 treatment.

### Effects of rhBMP-2 on gene expression profiling

**Figure 3:**
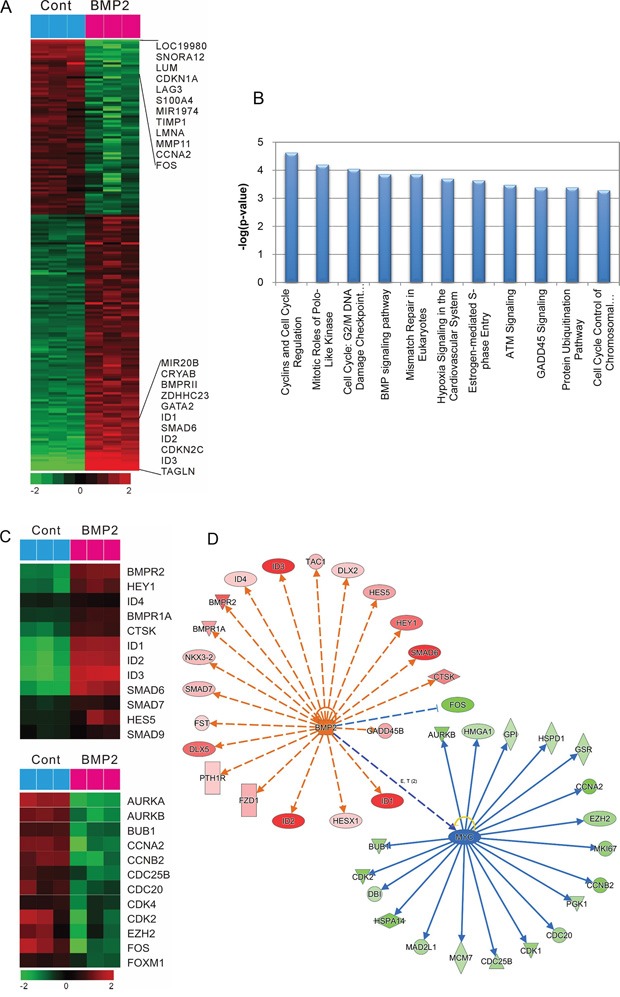
Gene expression profile regarding the effect of rhBMP-2 in SNU484 gastric cancer cells **A.** A microarray heat map summarizing the effect of rhBMP-2 using an Illumina bead array platform. Data are presented in a matrix format. Rows represent the individual genes and the columns represent samples. Red, up-regulation; green, down-regulation. **B.** Top canonical pathway analysis for different functional categories. **C.** Microarray heat map summarizing BMP signaling-related genes by rhBMP-2 treatment (top) and cell cycle regulation signaling-related genes by rhBMP-2 treatment (down). Potential targets of BMP-2 and c-Myc. **D.** Gene networks of BMP-2 and c-Myc from an Ingenuity TM pathway analysis. Red, up-regulation; green, down-regulation; blue, predicted inhibition; orange, predicted activation. Orange dash line, leads to activation; blue line, leads to inhibition.

To test whether the regulation of gene expression mediated the BMP-induced human gastric cancer cell death, microarrays were used to identify gene expression patterns using an Illumina bead array platform. The data showed that more than 1453 genes were regulated by BMP-2 treatment. Specifically, the expression of 826 genes was significantly upregulated, and 609 genes were significantly downregulated after the treatment of SNU484 cells with rhBMP-2 (Figure 3A). Gene ontology (GO) and canonical pathway analysis to determine the biological characteristics of selected genes revealed that cell cycle regulation, the mitotic roles of polo-like kinase, G2/M damage checkpoint regulation, and the BMP signaling pathway were controlled by treatment with rhBMP-2 (Figure 3B). To evaluate the association between BMP-2 treatment and signaling pathway associations, a further gene-network analysis was performed using Ingenuity™ Pathway Analysis. We found that more than 20 networks were enriched for cell cycle, cellular growth and proliferation, cell death, and survival. Furthermore, an upstream analysis of the network revealed that the BMP2 signaling pathway was highly activated as shown by BMP-2 downstream target genes *BMPRA*, *BMPR2*, *CTSK*, *ID1*, *ID2*, *ID3*, *ID4*, *HES5*, *HEY1*, *SMAD6*, *SMAD7*, and *SMAD9* (Figure 3C and 3D). *MYC* signaling was significantly downregulated after treatment with rhBMP-2 as shown by the downstream target genes *AURKB*, *CCNA2*, *CCNB2*, *CDC20*, *CDC25B*, *CDK1*, *CDK2*, *EZH2*, and *FOXM1* (Figure 3C and 3D). Therefore, *MYC* may be the core gene responsible for regulating many downstream genes following rhBMP-2 treatment.

### Effect of rhBMP-2 on Myc signaling on gastric cancer cells

**Figure 4:**
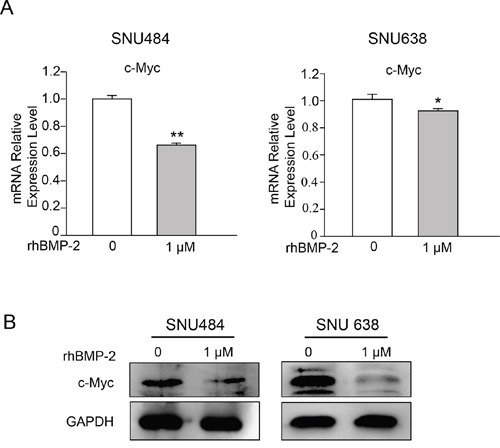
Validation of c-Myc expression following the treatment of gastric cancer cells with rhBMP-2 **A.** c-Myc mRNA by qRT-PCR and **B.** western blots after treatment of SNU484 and SNU638 cell lines with rhBMP-2 for 72 h. Expression of the target genes monitored and normalized by GAPDH expression. The ratios of normalized expression of target genes relative to control (untreated) cells are shown. Results are presented as the means ± SEM.

To determine whether BMP-2 expression was inversely correlated with *MYC* expression, we further investigated c-Myc expression that was decreased by rhBMP treatment in the microarrays using RT-PCR and western blot. RT-PCR revealed that 1 μM rhBMP-2 treatment significantly decreased *c-Myc* mRNA levels (Figure 4A). Additionally, treatment with rhBMP-2 significantly attenuated c-Myc protein expression (Figure 4B). These data suggest that rhBMP-2 inhibits *c-Myc* expression in gastric cancer cells. These observations are in agreement with the microarray data, suggesting that rhBMP-2 may suppress the expression of c-Myc in gastric cancer cells.

### Cell cycle regulatory protein expression by rhBMP-2

**Figure 5:**
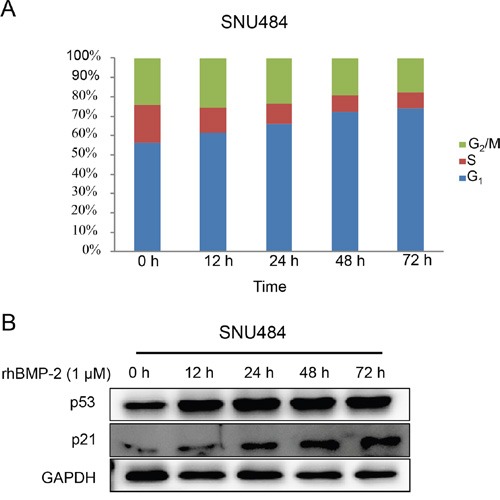
Cell cycle distribution of gastric cancer cells by rhBMP-2 **A.** Cells were incubated with 1 μM rhBMP-2 for 0, 12, 24, 48, or 72 h. Cell cycle distribution was evaluated as a percentage of cells in the G1, S, and G2/M phase. **B.** Protein measurements for p53 and p21 following treatment of rhBMP-2 in gastric cancer cells for 0, 12, 24, 48, or 72 h. Target gene expression was monitored and normalized to GAPDH expression.

The microarray data revealed that the regulation of the cell cycle was induced by rhBMP-2 treatment. To determine the involvement of rhBMP-2 in cell cycle regulation, we determined the cell cycle distribution using a fluorescence-activated cell sorting analysis. Cell cycle fractions were measured at 0, 12, 24, 48, and 72 h after treatment of the SNU484 cells with rhBMP-2. The population in the G1 phase of the cell cycle significantly and time-dependently increased (Figure 5A). Since BMP-2 induced G1 cell cycle arrest in gastric cancer cells, we tested the cells for alterations in cell cycle regulatory proteins (e.g., p21 and p53) after treatment with rhBMP-2. We found that p53 protein levels increased at 12 h and continued increasing until 72 h. Protein levels of p21 we also increased at 24 h after rhBMP-2 treatment and gradually increased until 72 h. These results suggest that rhBMP-2 induced cell cycle arrest and thus, inhibited gastric cell proliferation.

### Effect of rhBMP-2 on AURKA and AURKB signaling in gastric cancer cells

**Figure 6:**
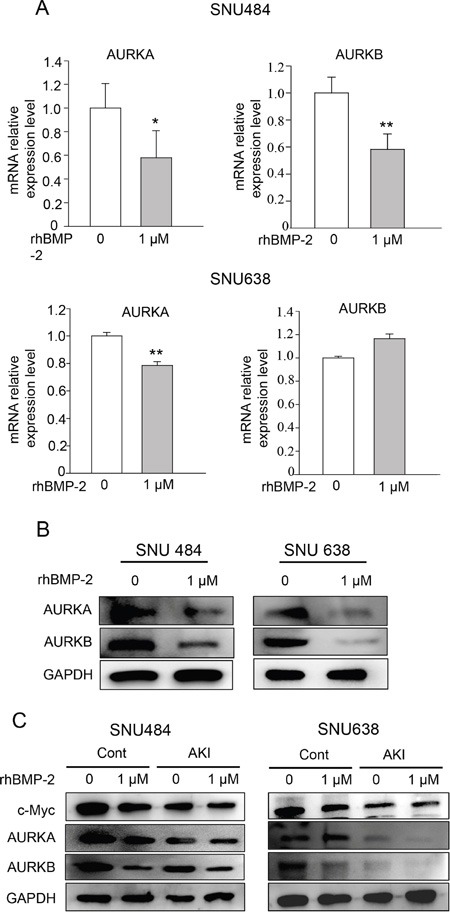
Validation of AURKA and AURKB expression after gastric cancer cell treatment with rhBMP-2 AURKA and AURKB mRNA determined by **A.** qRT-PCR and **B.** western blots after treatment of SNU484 and SNU638 cell lines with rhBMP-2 for 72 h. The expression of target genes was monitored and normalized to GAPDH expression. The ratios of the normalized expression of target genes relative to the control (untreated) cells are shown. Results are expressed as the means ± SEM. **C.** Western blotting assays for the expression of c-Myc, AURKA, and AURKB proteins in aurora kinase A and B inhibitor pretreated cells treated with or without 1 μM rhBMP-2 for 48 h in SNU484 and SNU638 cell lines. GAPDH was used as an internal control.

We next determined the expression of aurora kinase A *(AURKA*) and aurora kinase B (*AURKB*), which are downstream target genes of Myc. We found that both target genes were decreased by rhBMP treatment in the microarray experiments. Treatment with rhBMP-2 significantly decreased the mRNA levels of *AURKA* and *AURKB* in SNU 484 cells (Figure 6A). RhBMP-2 significantly decreased *AURKA* mRNA but no significant difference was observed for *AURKB* after rhBMP-2 treatment of SNU638 cells. In western blots, similar observations were found for both types of gastric cancer cells. Moreover, rhBMP-2 suppressed AURKA and AURKB protein levels in the SNU484 and SNU638 cells (Figure 6B). To further investigate whether rhBMP-2 regulates c-Myc expression mediated through AURK in gastric cancer cells, we used an AURK inhibitor (500 nM) in combination with rhBMP-2 in SNU484 and SNU638 cells. As shown in Figure 6C. AURKA and AURKB were suppressed following treatment with the AURK inhibitor. Moreover, the AURK inhibitor suppressed c-Myc protein expression in the SNU484 and SNU638 cells. In addition, the AURK inhibitor combined with the rhBMP-2 treatment significantly decreased the expression of c-Myc expression levels compared to rhBMP-2 or AURK inhibitor treatment alone in SNU484 and SNU638 cells. These results indicate that the rhBMP-2-induced decrease in c-Myc expression was mediated by AURK in gastric cancer cells.

### Effect of rhBMP-2 on β-catenin signaling in SNU484 and SNU638 cells

**Figure 7:**
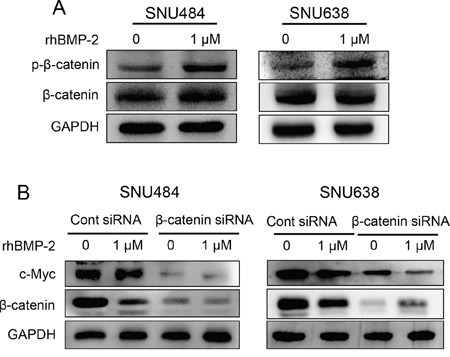
Validation of β-catenin expression after treatment of gastric cancer cells with rhBMP-2 **A.** β-catenin protein expression by western blot after treatment of SNU484 and SNU638 cells with rhBMP-2 for 72 h. **B.** Western blotting assays for the expression of c-Myc, p-β-catenin, and β-catenin proteins in β-catenin siRNA transfected cells treated with or without 1 μM rhBMP-2 for 48 h in SNU484 and SNU638 cell lines. GAPDH was used as an internal control.

The β-catenin signaling pathway is important for cancer progression, and the overexpression of β-catenin has been reported for various cancer types, including gastric cancer [22]. To further investigate the interaction between rhBMP-2 and the β-catenin signaling pathway, we measured the β-catenin protein levels after rhBMP-2 treatment using western blot. The level of phosphorylated β-catenin protein was increased in both cell lines (Figure 7A). However, no change was observed in the level of β-catenin protein with rhBMP-2 treatment (Figure 7A). These data suggest that the β-catenin oncogene was significantly downregulated by the rhBMP-2 treatment of gastric cancer cells. To further investigate whether rhBMP-2 regulates c-Myc expression mediated through β-catenin in gastric cancer cells, we performed a β-catenin siRNA experiment with rhBMP-2 treatment. The silencing of β-catenin inhibited c-Myc protein expression in the SNU484 and SNU638 cells (Figure 7B). Furthermore, combination treatment of rhBMP-2 with the silencing of β-catenin significantly decreased the expression of c-Myc compared to rhBMP-2 treatment or silencing of β-catenin alone in SNU484 and SNU638 cells. These results suggest that the rhBMP-2-induced decrease in c-Myc expression was mediated through β-catenin in gastric cancer cells.

## DISCUSSION

The aim of this study was to investigate the biological function of rhBMP-2 on the gene expression profile in human gastric cancer cells. RhBMP-2 significantly suppressed the proliferation of gastric cancer cells by changing the expression of genes involved in the progression and regulation of the cell cycle. We found that rhBMP-2 significantly downregulated c-Myc transcription activity in gastric cancer cells.

Gene expression profiling has been used to identify biomarkers for the purpose of clarifying biological mechanisms [5, 23], categorizing cancer subtypes [5, 24], predicting cancer prognosis [5, 25], and elucidating gene expression in response to drugs [5, 26]. Since the safety issues concerning rhBMP-2 treatment have been debated since the beginning of its application, the effects of exogenous BMP-2 have been explored in many studies. In particular, gene expression profiling has been used to identify alterations in various signaling pathways by rhBMP-2 both *in vitro* and *in vivo* [27–30]. For example, Liu et al. reported that BMP-2 promotes the differentiation of osteoblasts and chondroblasts in RUXN2-deficient cell lines, and Zhou et al. reported that rhBMP-2 affects the gene expression of the TGF-beta/BMP pathways in human dermal fibroblasts [27, 28]. Zou et al. used a microarray analysis to demonstrate that rhBMP-2 recruits progenitor cells and promotes the proliferation and differentiation during anterior lumbar interbody fusion [31]. In addition, head and neck squamous cell carcinomas with high baseline BMP-2 protein levels are associated with higher rates of local recurrence [29]. This result supports the use of rhBMP-2 in tissue-engineering reconstructive approaches for cancer-related defects [29]. Although studies have been conducted regarding rhBMP-2 functions in the context of cancer [10, 14, 21, 29], the precise, comprehensive, and functional mechanisms through which rhBMP-2 affects gastric cancer cells remain unknown. In this study, we used gene expression profiling to uncover the molecular mechanisms underlying the functions of rhBMP-2 in gastric cancer cells. We discovered that the expression of 1453 genes was increased or decreased at least twofold following rhBMP-2 treatment. Among these genes, 826 were increased and 609 were decreased in expression. Based on the GO analysis, many of these genes were enriched in cyclins and cell cycle regulation, mitotic roles of polo-like kinase, and G2/M DNA damage checkpoint regulation. We also found that rhBMP-2 treatment induced genes associated with the BMP-2 signaling pathway. In agreement with these microarray data, we found that rhBMP-2 stimulated the expression of BMP-2, BMPRI, and BMPRII proteins.

Network upstream analysis revealed that rhBMP-2 significantly suppressed the expression of *MYC* and its downstream target genes *AURKA*, *AURKB*, *CCNA2*, *CCNB2*, *CDC20*, *CDC25B*, *CDK1*, *CDK2*, *EZH2*, and *FOXM1*. This downregulation of *MYC* signaling might be responsible for the inhibition of gastric cancer cell growth since we found that rhBMP-2 significantly suppressed SNU484 and SNU638 gastric cancer cell viability by MTT and soft agar colony formation assays. We validated *c-Myc* expression after treatment with rhBMP-2 by qPCR and western blot assay; we found that c-Myc expression was significantly suppressed by rhBMP-2 treatment in both types of gastric cancer cells. Myc is recognized as a crucial oncogene in many types of human cancers, including gastric cancer [32–34]. Therefore, the downregulation of c-Myc by rhBMP-2 might be responsible for the inhibition of gastric cancer cells.

In addition, our data showed that rhBMP-2 stimulated a significant, time-dependent increase in the proportion of the cell population in G1 phase of the cell cycle in SNU484 cells. P53 and p21 expression were gradually increased after treatment with rhBMP-2. These results indicate that rhBMP-2 might regulate cell cycle arrest and thus, inhibit gastric cell proliferation. A study of AURKA and regulation of the cell cycle in cancer through multiple signal pathways found that AURKA diminished pRb, p53, P21waf1/cip1, and p27cip/kip but increased Plk1, CDC25, CDK1, and cyclin B1 to promote cell cycle progression [35]. Overexpression of AURKA inhibits p53 and p73 levels in cancer cells [32, 36, 37]. AURKA and AURKB are also overexpressed in a variety of human cancers, and the activation of AURKA and AURKB has oncogenic effects [38–42]. Therefore, both AURKA and AURKB appear to be associated with malignant phenotypes of cancer and function as oncogenes. We found that rhBMP-2 decreased mRNA and protein expression of *AURKA* and *AURKB*, and this may be linked to increased levels of p53 and p21 in gastric cancer cells, suggesting that rhBMP-2 arrests the cell cycle in gastric cancer cells. Furthermore, we assumed that the down-regulation of c-Myc by rhBMP-2 seemed to be correlated with AURK in gastric cancer cells. To test the hypothesis that the down-regulation of c-Myc is mediated through AURK by rhBMP-2 in gastric cancer cells, we measured the expression of c-Myc using an AURK inhibitor with rhBMP-2 treatment. An AURK inhibitor decreased c-Myc expression levels in gastric cancer cells. In addition, combination treatment with an rhBMP-2 and AURK inhibitor significantly decreased c-Myc expression in SNU484 and SNU638 cells compared to treatment of the rhBMP-2 or AURK inhibitor alone. Several studies have reported that Myc and AURKA directly regulate each other at the transcriptional level in various cancer cells [32, 43, 44]. For instance, AURKA stimulates telomerase activity by upregulating Myc in human ovarian and breast epithelial cells [43]. Destruction of AURKA or Myc inhibits the malignant phenotypes of hepatocellular carcinoma cells [32]. In agreement with previously published articles, our results showed that there was a direct correlation with c-Myc expression and AURK, and both were suppressed by rhBMP-2 in gastric cancer cells. Therefore, our results suggest that the down-regulation of c-Myc by rhBMP-2 may be mediated through AURK, the downstream target genes of c-Myc, and induce the inhibition of the cell cycle.

We also presented evidence that rhBMP-2 inhibited the activation of β-catenin in the gastric cancer cells SNU484 and SNU638. We found that rhBMP-2 increased phosphorylated β-catenin in gastric cancer cells. β-catenin is regarded as a downstream target of Wnt signaling and an upstream target of c-Myc [45, 46]. β-catenin is also known as an oncogene in human gastric cancer [46]. β-catenin regulates the expression of c-Myc and the cell cycle. From our data, silencing of β-catenin was found to significantly suppress c-Myc expression in gastric cancer cells. Moreover, the combination treatment of rhBMP-2 and β-catenin silencing significantly decreased c-Myc expression in gastric cancer cells compared to the treatment of rhBMP-2 alone or silencing of β-catenin alone. These results suggest that rhBMP-2 inhibits β-catenin and c-Myc expression in gastric cancer cells. Taken together, these findings support the hypothesis that rhBMP-2 suppresses the activation of the Wnt signaling pathway with their target genes and thus, causes gastric cancer cell death.

In summary, our findings indicate that rhBNP-2 suppresses gastric cancer cell proliferation by inhibiting β-catenin and c-Myc with AURKA and AURKB expression to regulate inhibition of the cell cycle. RhBMP-2 may also be used as a therapeutic agent in gastric cancer. Further studies of rhBMP-2 in many cancers are needed because it is reported to have different effects on cell proliferation or invasion of human cancer. However, our data suggest that rhBMP-2 could be safely administered to patients who received gastric or gastroesophageal cancer surgery.

## MATERIALS AND METHODS

### Reagents and antibodies

Recombinant human BMP-2 was obtained from Dae Woong Pharmaceuticals (Seoul, South Korea). The BMP-2 antibody was aquired from Abcam (Cambridge, UK) and p-Smad1/5/8, β-catenin, p-β-catenin, ERK1/2, and p-ERK1/2 antibodies were purchased from Cell Signaling Technology (Danvers, MA, USA). Antibodies for BMPRII, c-Myc, AURKA, AURKB, and GAPDH were from Santa Cruz Biotechnology (Santa Cruz, CA, USA). β-catenin small interfering RNA (siRNA) was purchased from Santa Cruz Biotechnology (Dallas, TX, USA) and the aurora kinase A and B inhibitor (AURK inhibitor) was obtained from TOCRIS Bioscience (Bristol, UK).

### Cell cultures and viability measurements

Human gastric cancer cell lines SNU484 and SNU638 were obtained from the Korean Cell Line Bank (Seoul National University, South Korea). SNU484 and SNU638 cells were maintained in RPMI1640 medium containing 10% fetal bovine serum and 100 μg/mL penicillin and 100 μg/mL streptomycin (all from Gibco-BRL, Grand Island, NY, USA) at 37°C in a humidified incubator with 5% CO_2_. Cell viability was assessed by 3-4,5-dimethylthiazol-2,5-diphenyltetrazolium bromide (MTT) assays as described previously [7]. SNU484 and SNU638 cells were seeded at 10^4^ cells/well in 96-well plates and allowed to adhere. After 24 h, rhBMP-2 was added to 10, 250, 500, or 1000 nM for 72 h. At the end of the culture period, 50 μL of 2 mg/mL MTT was added to the medium and incubated for 3 h at 37°C with 5% CO_2_. Dimethyl sulfoxide (200 μL/well) was added to dissolve the formazan crystals. The measurement was obtained at 570 nm using an Epoch Microplate Reader (Bio-Tech, Winooski, VT, USA). All results are presented as a percentage compared to the untreated cells.

### Small interfering RNA transfection and treatments

β-catenin small interfering RNA (siRNA) or control siRNA (Santa Cruz Biotechnology) (100 pM) were transfected into SNU484 and SNU638 cells (2 × 10^5^ cells/well) using Lipofectamime 2000 (Invitrogen, Pittsburgh, PA, USA) according to the manufacturer's instructions. At 24 h after transfection, the cells were used for further treatment. The SNU484 and SNU638 cells were treated with an AURK inhibitor (TOCRIS Bioscience, Bristol, UK, 500 nM), and after 24 h the cells were used for further treatment.

### Cell cycle analysis

Cell cycle regulation was measured as previously described [47]. Briefly, the cells were plated into 100 mm dishes at 1 × 10^6^ cells in RPMI 1640. The cells were treated with 1 μM rhBMP-2 for 0, 12, 24, 48, or 72 h and then stained with propidium iodide (Sigma Chemicals, St. Louis, MO, USA). The percentage of cells was measured with a FACStar flow cytometer (Becton-Dickinson, San Jose, CA, USA) and analyzed using Becton-Dickinson software (Lysis II, Cellfit) as described previously [48].

### Soft agar colony formation assays

Colony formation assay in soft agar was assessed as described previously [7, 49]. Briefly, the bottom layers of the agarose gel consisted of 2 mL serum-supplemented medium and 0.4% agar. Top layers contained 1 mL serum-supplemented medium and 0.35% agar containing 2.5 × 10^4^ cells with or without 1 μM rhBMP-2. Cells were incubated at 37°C in a humidified 5% CO_2_ atmosphere for four weeks. Colonies were counted under light microscopy and photographed.

### RNA isolation, microarray experiments, and gene expression data

Total RNA was extracted using MirVana™ miRNA isolation labeling kits (Ambion Inc., Austin, TX, USA) in accordance with the manufacturer's protocol. Microarrays were performed as described previously [50]. The total RNA (500 ng) was used for labeling (Total Prep RNA amplification kit, Ambion Inc.) and hybridization (Illumina Human-12 BeadChip V.4 microarray, Illumina, San Diego, CA, USA) in accordance with the manufacturer's protocols. Gene expression data were extracted using GenomeStudio software (Illumina, San Diego, CA, USA), and the data were normalized using the quantile normalization method in the Linear Models for Microarray data package in the R language environment (http://www.r-project.org). Cluster and Treeview programs were used to generate heat maps of the gene expression data [51]. A network analysis used the Ingenuity Pathways Analysis software (Ingenuity Systems Inc., QIAGEN Silicon Valley, CA, USA). Microarray studies were performed by the Shared Research Equipment Assistance Program, Korea Basic Science Institute, MEST.

### Real-time RT-PCR

A real-time RT-PCR analysis was performed as described previously [52]. Briefly, a total of 1 μg RNA was reverse transcribed in 20 μL reactions using PrimeScript RT reagent kits (TaKaRa, Japan) according to the manufacturer's protocol. Real-time PCR was performed in 10 μL reactions (1 μL cDNA, 5 μL 2x SYBR Premix Ex Taq II, 0.4 μL each 10 μmol/L forward and reverse primers, 0.2 μL Rox Reference Dye, and 3 μL H_2_O) using an ABI Prism 7900 Sequence Detection System (Applied Biosystems, Foster City, CA, USA). The PCR program was 30 s at 95°C, 15 s at 95°C and 1 min at 60°C for 40 cycles. Data analyses were performed using the comparative Ct method with normalization to GAPDH expression for each sample. The following primers were used to amplify each gene:

BMP-2: sense 5′-TCAAGCCAAACACAAACAGC and antisense 5′- AGCCACAATCCAGTCATTCC;

c-Myc: sense 5′-CAGCTGCTTAGACGCTGGATT and antisense 5′-GTAGAAATACGGCTGCACCGA-3′;

Aurora A: sense 5′-GAATGCTGTGTGTCTGTCCG and antisense 5′-GCCTCTTCTGTATCCCAAGC;

Aurora B: sense 5′-GATGACTTTGAGATTGGGCG and antisense 5′-GGGACTTGAAGAGGACCTTG.

### Western blotting

Western blots were performed as described previously [49, 50]. Briefly, the cells were seeded at 1 × 10^6^ cells in 10 cm culture dishes. After 24 h, cells were treated with or without 1 μM rhBMP-2 for 72 h. Cells were washed with PBS and harvested. Pellets were lysed in ice-cold PRO-PREP™ (iNtRON Biotechnology, Seoul, South Korea), and the extracts were incubated on ice for 30 min and centrifuged at 13,200 rpm for 20 min at 4°C. The supernatants were collected and the protein concentration determined using BSA protein assay kits (Pierces Biotechnology, Inc., Rockford, IL, USA). The proteins were separated by 10% sodium dodecyl sulfate polyacrylamide gel electrophoresis (SDS-PAGE) and transferred to PVDF membranes (GE Healthcare Life Sciences, Buckinghamshire, UK). After blocking with 5% skim milk for 1 h, the blots were probed with primary antibodies against BMP-2, BMPRII, p-Smad1/5/8, β-catenin, p-β-catenin, ERK1/2, p-ERK1/2, c-Myc, Aurka, or Aurkb overnight followed by incubation with secondary antibody-horseradish peroxidase conjugate for 1 h. Immunoreactivity was detected using chemiluminescence kits (Amersham, Arlington Heights, IL, USA).

### Statistical analysis

Each experiment was repeated more than three times. The results represent the means ± standard error of the mean (SEM). Comparisons among the groups were performed using a one-way ANOVA with a Student's *t*-test. P < 0.05 was considered to be statistically significant.

## SUPPLEMENTARY MATERIALS FIGURE


