# Supplementary material for: Recombinant human bone morphogenetic protein-2 inhibits gastric cancer cell proliferation by inactivating Wnt signaling pathway via c-Myc with aurora kinases
Source: Oncotarget. 2016 Sep 12;7(45):73473–85. doi: 10.18632/oncotarget.11969 (PMC5341992; doi:10.18632/oncotarget.11969)
Supplement: Supplementary file 1 [file oncotarget-07-73473-s001.pdf]

Recombinant human bone morphogenetic protein-2 inhibits gastric cancer cell proliferation by inactivating Wnt signaling pathway via c-Myc with aurora kinases

SUPPLEMENTARY FIGURE

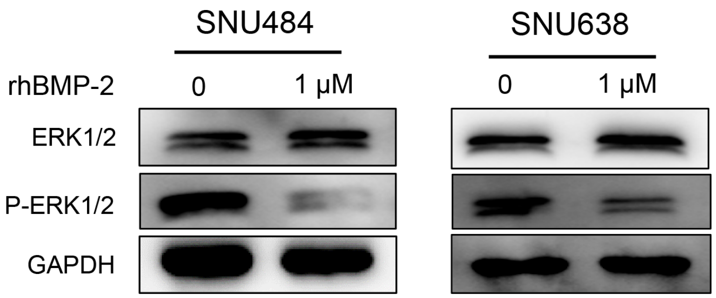

Supplementary Figure S1: Validation of ERK proteins after treatment with rhBMP-2 in gastric cancer cells. ERK1/2 and p-ERK1/2 were measured by western blotting of SNU484 and SNU638 cells after treatment with rhBMP-2 for 48 h and immunoblotting with the indicated antibodies. GAPDH was used as an internal control.
